# Editorial: Women in primary immunodeficiencies

**DOI:** 10.3389/fimmu.2023.1268595

**Published:** 2023-10-12

**Authors:** Sara Sebnem Kilic

**Affiliations:** Department of Pediatric Immunology-Rheumatology, Faculty of Medicine, Bursa Uludag University, Bursa, Türkiye

**Keywords:** immunology, immunodeficiency, vaccine, women, award, inborn error immunity

Women scientists have made many contributions to the scientific arena regarding human health and diseases. They have made great efforts to reveal the secrets in the science of immunology, developing new experimental methods and showing effective treatment alternatives. While focusing on the mysteries of the defense systems of many living organisms in nature, women scientists have adopted the principle of pursuing their vital interests. Women scientists have made significant advances in genetics, molecular biology, microbiome, genomics, imaging, responses to infectious diseases, drug development, and identification of genetic disorders underlying primary and secondary immunodeficiencies (1–4). We are proud to have outstanding female immunologists who have made significant strides in autoimmunity, cancer immunology, vaccines, allergy, and the importance of epithelial barriers, cellular and systems biology, too many to count.

Increasingly, women have risen to leadership positions in areas such as laboratory supervisors, study protocol leaders, institute directors, university rectors, foundation and association presidents, and pharmaceutical company CEOs.

Of the more than 700 laureates of a Nobel, only 30 have been received by women. The first woman to receive a Nobel Prize was Marie Curie with her spouse for their work on spontaneous radiation. Lastly, Tu Youyou received Nobel Prize in Physiology or Medicine in 2015. She demonstrated the effectiveness of Artemisinin in malaria cases resistant to chloroquine treatment, and it is thought that she signed one of the most critical pharmacological discoveries of half a century because of this success.

Rosalind Elsie Franklin (1920 –1958) was a British chemist whose x-ray diffraction studies provided crucial clues to the structure of DNA and quantitatively confirmed the Watson-Crick DNA model. Her work was essential to understanding the molecular structures of DNA, RNA, viruses, coal, and graphite. She photographed the double helix structure of DNA. Unfortunately, Franklin’s contributions to the discovery of the structure of DNA were largely unrecognized during her life. It has been suggested that Franklin would have ideally been awarded a Nobel Prize in Chemistry, along with Wilkins, after her death. Throughout her 16-year career, Franklin published steadily: 19 articles on coals and carbons, five on DNA, and 21 on viruses.

Barbara McClintock (1902 –1992) was an American scientist and cytogeneticist who was awarded a Nobel Prize in Physiology or Medicine in 1983. She demonstrated the role of the telomere and centromere, regions of the chromosome that are important in the conservation of genetic information. Her work has been considered one of the top two great discoveries in genetics.

Rita Levi-Montalcini (1909-2012) was an Italian neurobiologist. She was awarded the 1986 Nobel Prize in Physiology or Medicine jointly with colleague Stanley Cohen for the discovery of nerve growth factor (NGF) in 1986. NGF was the first of many cell-growth factors found in animals’ bodies, causing tumors. It plays an essential role in the growth of nerve cells and fibers in the peripheral nervous system. She held the position of Director of the Institute of Cell Biology of the Italian National Council of Research, From 1969 to 1978 (1).

Gender imbalance in science is a long-standing problem that has attracted attention in recent years. While there have been significant gains in gender equality in recent years, progress is slow and not taking place at the required pace and scale. In particular, the gender gap persists in education, politics, and health (5–7).

Data from international immunology meetings show a very low proportion of women among symposia and keynote speakers. Additionally, while the presence of women as first authors of articles has increased significantly in recent years, their representation as senior writers has yet to progress at the same rate. Such disparities are most remarkable in high-impact journals. These differences can lead to the underrepresentation of women at higher levels of science (8).

The Gender Social Norms Index (GSNI) assesses people’s attitudes toward women’s roles in various areas of life in four key dimensions: political, educational, economic, and physical integrity. The 2023 GSNI contains data from 80 countries and territories of the World Values Survey, accounting for 85 percent of the global population. According to the GSNI, the percentage of people with biased gender and dimension is 88.45 in Turkey; however, 27.15 in the UK and 50.69 in the USA. Therefore, geography is an important factor affecting the success of women in the field of business. A woman living in the Eastern and middle eastern geography should be able to enjoy the rights that women in the Western regions have so that they can carry out original works in different habitats in various parts of the world. Prejudiced gender concepts constitute a significant obstacle to achieving gender equality and supporting all women and girls in the social and scientific arena.

Turkish women scientists have accomplished successful works for the development of science in countries where laboratory and scientific research opportunities are more comprehensive than in their own countries (9, 10).

Özlem Türeci is a German physician, scientist and entrepreneur of Turkish origin. She co-founded the biotechnology company BioNTech, which in 2020 developed the first messenger RNA-based vaccine approved for use against COVID-19 (11, 12). She has been Professor of Personalized Immunotherapy at the Helmholtz Institute for Translational Oncology and Johannes Gutenberg University Mainz since 2021. Dr Türeci has won a number of awards, including the Princess of Asturias Awards, the Georges Köhler Prize, and the Paul Ehrlich and Ludwig Darmstaedter Prize (11, 12).

Another Turkish origin scientist Mübeccel Akdis has been working at the Swiss Institute of Allergy and Asthma Research (SIAF) since 1995 and became a professor in 2015. She contributed to understanding the mechanism of skin allergies and atopic dermatitis and played a leading role in elucidating the tolerance mechanisms. Dr. Akdis has worked as a group leader of immune regulation at SIAF. Her primary research contributions are on mechanisms of allergen tolerance, high-dose allergen exposure models in humans, allergen-specific immunotherapy mechanisms, T regulatory cells’ functions, NK regulatory cells, and IL-10-producing human B regulatory cells (13, 14). She received numerous awards, including Ferdinand Wortman Prize, Professor Hans Storck Award, Sedat Simavi Medicine Award, and Paul Ehrlich Award 2020 for Experimental Research.

Dr. Gulbu Uzel is a clinical immunologist with expertise in a national and international reputation in the area of human immunodeficiency disorders from Türkiye. Her research and clinical work targeted to define the mechanisms of immune dysregulation for the inborn errors of immunity and to describe new genes altering immune responses in patients with systemic autoimmunity. She has worked at the NIH and initiated, founded, and set up the Primary Immunodeficiency Clinic at the NIH, and has discovered and described two critical human defects: PIK3CD GOF mutations leading to lymphoproliferation and immunodeficiency and CTLA-4 haploinsufficiency leading to immune dysregulation and autoimmunity (15, 16).

To celebrate the scientific achievements and discoveries of the world’s pioneer female immunologists, we feature reviews that cover the latest research and advances in immunology written by leading female scientists. With this Research Topic, the section on Primary Immunodeficiency of Frontiers in Immunology offered female scientists the opportunity to promote their work by publishing original studies or reviews that address challenging issues in the field of inborn errors of immunity.

Despite various challenges and obstacles, women in science have made significant contributions throughout history. The successes of influential personalities such as Marie Curie or Özlem Türeci will pave the way for the next generation of women in scientific fields. Their existence and the strength of their struggle continue to inspire women in scientific disciplines worldwide.

To sum up, women have contributed in many areas, from demonstrating immune cell differentiation and function to developing innovative technologies and creating new algorithms in diagnosis and treatment. The increase of successful women in science and medicine in recent years is a great honor for societies. While progress has been made in overcoming these obstacles over the years, much work still remains to be done to achieve gender equality in science. Special projects and foundations are needed to increase the visibility of female immunologists. Platforms of female immunologists can be helpful to ensure that women are nominated as speakers, chairpersons, and committee members at immunology-related meetings and the editorial boards of journals as experts in decision-making bodies. The presence of leading female authors in the fields of immunology and immunodeficiency in recent years is a source of pride for their countries and societies. For this reason, projects that will highlight the role models of such women and enable them to be recognized by the community should be implemented in a short time.

## Author’s note

History has shown with examples of the difficulties experienced by all women who contribute to the scientific arena and that they should make more efforts in this field than their male colleagues. In this article, I tried to give examples of women who received awards for their work or who shed light on immunology with their groundbreaking innovations. Some suggestions were made about what to do in order for women to show themselves in the field of science, and ideas were put forward on how to pave the way for women.

## Author contributions

SK: Writing – original draft.
